# Oxidative and Nitrosative Stress in Age-Related Macular Degeneration: A Review of Their Role in Different Stages of Disease

**DOI:** 10.3390/antiox10050653

**Published:** 2021-04-23

**Authors:** Caterina Toma, Stefano De Cillà, Aurelio Palumbo, Divya Praveen Garhwal, Elena Grossini

**Affiliations:** 1 Eye Clinic, University Hospital Maggiore Della Carità, 28100 Novara, Italy; caterina.toma@maggioreosp.novara.it (C.T.); stefano.decilla@med.uniupo.it (S.D.C.); aurepa95@gmail.com (A.P.); 2 Department of Health Sciences, University East Piedmont “A. Avogadro”, 28100 Novara, Italy; 3 Laboratory of Physiology and Experimental Surgery, Department of Translational Medicine, University East Piedmont “A. Avogadro”, 28100 Novara, Italy; divya.praveen@uniupo.it

**Keywords:** age-related macular degeneration, oxidative stress, nitrosative stress, mitochondrial function, autophagy, anti-vascular endothelial growth factor agents

## Abstract

Although the exact pathogenetic mechanisms leading to age-related macular degeneration (AMD) have not been clearly identified, oxidative damage in the retina and choroid due to an imbalance between local oxidants/anti-oxidant systems leading to chronic inflammation could represent the trigger event. Different in vitro and in vivo models have demonstrated the involvement of reactive oxygen species generated in a highly oxidative environment in the development of drusen and retinal pigment epithelium (RPE) changes in the initial pathologic processes of AMD; moreover, recent evidence has highlighted the possible association of oxidative stress and neovascular AMD. Nitric oxide (NO), which is known to play a key role in retinal physiological processes and in the regulation of choroidal blood flow, under pathologic conditions could lead to RPE/photoreceptor degeneration due to the generation of peroxynitrite, a potentially cytotoxic tyrosine-nitrating molecule. Furthermore, the altered expression of the different isoforms of NO synthases could be involved in choroidal microvascular changes leading to neovascularization. The purpose of this review was to investigate the different pathways activated by oxidative/nitrosative stress in the pathogenesis of AMD, focusing on the mechanisms leading to neovascularization and on the possible protective role of anti-vascular endothelial growth factor agents in this context.

## 1. Introduction

Age-related macular degeneration (AMD) is a complex multifactorial retinal degenerative disease primarily affecting the macula and progressively leading to irreversible central vision loss. It is the first cause of blindness in the Western world, with an estimation of up to 18.6 million people being affected by the blinding stages of the disease by 2040 worldwide [1], though a consistent decline in blindness secondary to AMD has been observed from 1990 to 2020 thanks to the introduction of anti-vascular endothelial growth factor agents (VEGF) [2]. In 2013, the Beckman Initiative for Macular Research Classification Committee proposed a new classification of AMD, in which progression of disease was defined by the size of drusen, presence of retinal pigment epithelium (RPE) changes and occurrence of choroidal neovascularization (CNV), mainly driven by the over-expression of VEGF, and/or geographic atrophy (GA) in the late stages [3]. The Consensus on Neovascular AMD Nomenclature (CONAN) Study Group, based on information given by more recent imaging technologies such as optical coherence tomography (OCT) and OCT-angiography, proposed a new terminology for neovascularization and atrophy secondary to AMD [4]. In particular, the new term macular neovascularization (MNV) was introduced to replace the term CNV, as recent findings have shown that neovascularization (NV) not always originates from the choroid as previously reported [4].

The exact pathogenetic mechanisms leading to AMD are still not fully understood; however, it is well known that AMD is a multifactorial disease with multiple genetic and environmental factors contributing to its onset and progression [5,6,7,8,9,10,11]. Aging, the main risk factor for AMD, and other predisposing factors (e.g., cigarette smoking, high fat diet, excessive light exposure and genetic variants involving genes of the complement cascade such as Complement Factor H) significantly increase oxidative stress in the retina, leading to the excessive formation of reactive oxygen species (ROS), peroxidative processes and chronic inflammation [12,13,14,15,16,17,18,19,20], with an inability of the physiologic anti-oxidant local defensive mechanisms to counteract the accumulation of toxic metabolites, cellular debris and free radicals [21,22,23].

The present review will concentrate on the role of oxidative and nitrosative stress in the initiation and progression of AMD, with particular attention on the mechanisms leading to the formation of neovascularization in the context of AMD and the possible protective role of anti-vascular endothelial growth factor (VEGF) agents in this context.

## 2. The Role of Choriocapillaris (CC)/Bruch’s Membrane (BrM)/Retinal Pigment Epithelium (RPE)/Photoreceptor Complex in Different Forms of AMD

The maintenance of a physiologic relationship between the different components of the CC/BrM/RPE/photoreceptor complex is of primary importance to preserve its correct functioning and the breakdown of this equilibrium is involved in the changes occurring in AMD [24].

Summarizing the different contributions of these components to the local homeostasis, photoreceptors (and cones, in particular, which are the predominant photoreceptors in the macular region) are highly metabolically active cells rich in mitochondria (in their inner segments) and capable of phototransduction [25]. Their building and metabolism is supported by the RPE, which supplies nutrients for this purpose and plays a key role in maintaining the retinal homeostasis, serving as a limiting transport system between the outer retina and choroid [24,26] and as part of the waste degrading and recycling system of the retina, and photoreceptors in particular [24,27]. RPE cells are anatomically strictly connected to the BrM, which, thanks to its strategic location, regulates the diffusion of molecules between the RPE and choroid. Lastly, the CC, the capillary component of the choroid, provides all metabolic needs, including oxygen (O_2_), to photoreceptors from serum [25]. It emerges from this brief overview how the impairment of even a single component of the complex can become detrimental to the functioning of the whole system.

Even if it is well-established that the entire complex is altered in AMD, doubts remain on which could be the first component to be altered in the pre-clinical and early stages of disease. Photoreceptor degeneration and death, RPE changes (pigmentary changes, reduction in melanosomes and cell density and increase in lipofuscin granules), BrM thickening and formation of deposits and reduced density and diameter of the CC are all aspects seen in AMD. Post-mortem analyses performed with a specific image analysis technique on the human eyes of subjects affected by AMD allowed the correlation between the loss of RPE and the loss of CC [28], and a linear relationship between them was detected in GA [24]. Moreover, areas of RPE loss with underlying preserved CC were found at the edges of GA, suggesting that RPE might be the primarily affected site in GA [24,29]. In contrast, in neovascular AMD (nAMD) areas of significant CC reduction surrounding the NV were observed in association with areas of intact RPE, in this case indicating that the first trigger in nAMD could be the vascular impairment caused by an ischemic and/or inflammatory insult with secondary RPE dysfunction [24,30,31,32,33,34,35]. In a recent post-mortem study on human donor eyes, Lutty et al. showed the presence of significant CC dropout (measured as percent vascular area) in the macular region in eyes affected by early AMD compared to aged control eyes [36]. Moreover, they found, in two out of seven eyes with early AMD, the presence of hypercellular structures breaking through the BrM, which were considered as early forms of NV [36]. Seddon et al. had previously detected early NV in 40% of eyes with intermediate AMD [37]. As all areas of NV formation demonstrated an overlying complete RPE monolayer, it was hypothesized that CC dropout could lead to a hypoxic insult to RPE cells with overexpression of pro-angiogenetic factors, in particular VEGF, and a consequent stimulus to neo-angiogenesis [36,37,38].

## 3. Oxidative Stress as the First Trigger for AMD Initiation

**Figure 1:**
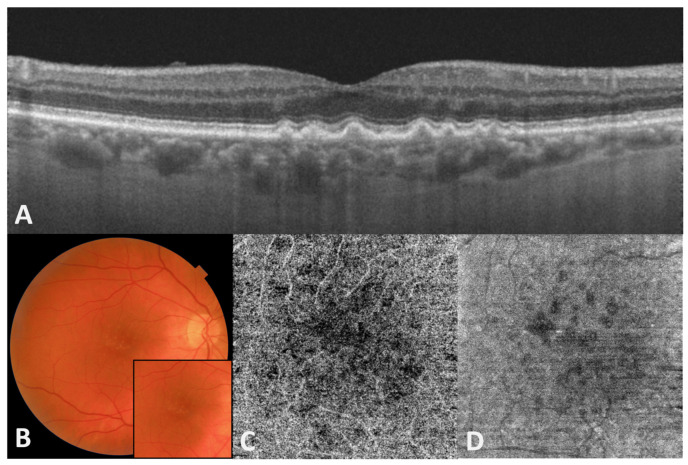
Right eye of an 86-year-old male patient with macular drusen. (**A**) Optical coherence tomography (OCT) B-scan centered on the fovea showing multiple drusen and no signs of exudation. (**B**) Color fundus photography of the posterior pole showing partially confluent small and intermediate drusen in the macular region. No hemorrhages or other signs of exudation are present. (**C**) OCT-angiography 4.5 × 4.5 mm macular slab segmented at the choriocapillaris level showing multiple areas of low signal due to reduced perfusion but no signs of macular neovascularization. (**D**) Corresponding en-face structural choriocapillaris image showing defects underlying drusen.

Non-neovascular AMD is the most frequent form of AMD and is defined by the presence of drusen, the first clinically detectable sign of the impairment of the CC/BrM/RPE/photoreceptor complex in AMD (Figure 1). Different in vitro models have been used to investigate the association of oxidative stress with the development of AMD and current knowledge supports the hypothesis of oxidative stress as the initial trigger for AMD pathogenesis [39,40,41]. Exposure of cultured primary fetal human RPE cells (hRPE) and ARPE-19 cells (a transformed cell line from the RPE of a 19-year-old male donor) to oxidative stress has been shown to increase the expression of several proteins involved in the processes of apoptosis, inflammation and DNA repair demonstrated in the pathologic changes typical of AMD [42,43,44,45,46,47,48]. Moreover, the use of hRPE cultures derived from adult stem cells from the RPE exposed to tert-butylhydroperoxide as a model of chronic oxidative stress demonstrated the upregulation of several proteins commonly found in drusen (such as αB-, βB1-, βB2-, βS- and βA4-crystallins, amyloid precursor protein, complement component 9 and VEGF-A) [49]. In addition, the development of subretinal deposits and RPE changes have been demonstrated in knock-out mouse models for antioxidant and toxic response genes such as the superoxide dismutase family (SOD-1 and 2), nuclear factor erythroid-derived 2-like 2 (NFE2L2 and Nrf2) and aryl hydrocarbon [50,51,52,53,54].

**Figure 2:**
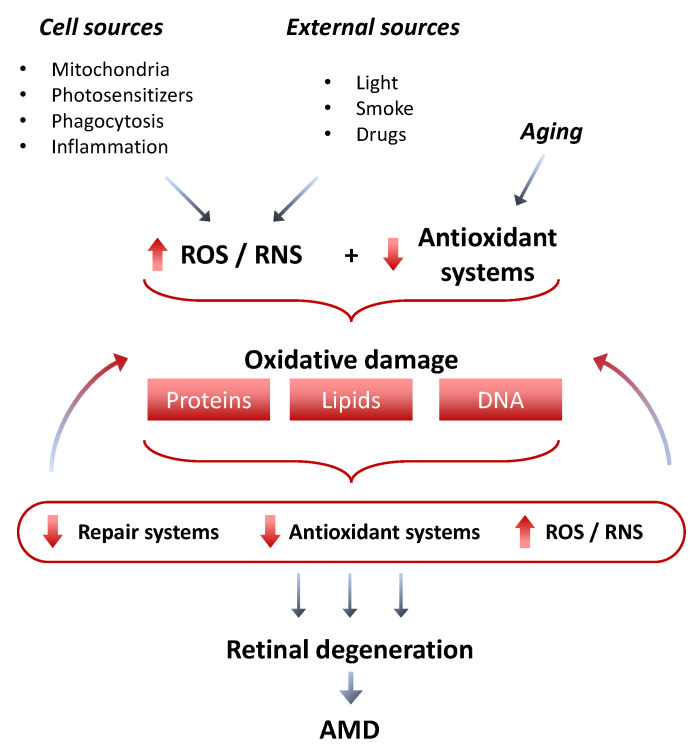
Mechanisms at the basis of oxidative damage in AMD. ROS: reactive oxygen species; RNS: reactive nitrogen species; AMD: age-related macular degeneration.

Retinal cells are particularly susceptible to oxidative damage due to their extremely high O_2_ consumption and metabolic activity and to their constant exposure to light and ultraviolet (UV) radiation; in particular, exposure to visible light (especially of shorter wavelength) has been associated with damage to photoreceptors and RPE cells (as UV radiation is mainly absorbed by the cornea and the lens) consequent to the local generation of high amounts of ROS, such as superoxide anion, hydroxyl radical, hydrogen peroxide and singlet oxygen, as byproducts of retinal metabolism [55,56,57,58,59,60,61]. Even if in moderate concentrations ROS play an important role in the regulation of protein function in the retina, at higher levels (as those produced and not counterbalanced during pathologic processes such as AMD) they have detrimental effects on cellular health, leading to the impairment of local homeostasis with the consequent activation of pathologic intra and intercellular pathways. Photoreceptors, cones in particular, and RPE cells are particularly susceptible to oxidative damage, being nonproliferative postmitotic cells lacking any system to detect DNA damage in their cell cycle checkpoints [62,63]. The main sources of ROS in AMD consist of: lipid peroxidation of phospholipid decosahexaenoic acid (DHA), one of the major components of photoreceptor membranes [64,65]; absorption of light by lipofuscin and the other photosensitizers present in the retina [66]; phagocytosis of the outer segments of photoreceptors by RPE cells with the consequent generation of hydrogen peroxide (H_2_O_2_) from NADPH oxidase in the phagosome; or β-oxidation of lipids in peroxisomes [23,67] (Figure 2). There are two different mechanisms hypothesized to be involved in lipofuscin-mediated ROS generation: the first is direct via interaction with light [68]; the second is mediated by the production of N-retinylidene-N-retinylethanolamine (A2E) [69]. Moreover, lipofuscin has also been demonstrated to be capable of decreasing the activity of lysosomal and antioxidant enzyme systems in the RPE [70]. Lipid peroxidation highly reactive end-products, such as 4-hydroxylnonenal (4-HNE), malondialdehyde (MDA), oxidized nucleotides and carboxyethyl pyrrole (CEP), have been demonstrated to be associated with drusen formation and RPE atrophic modifications in both human and animal eyes [19,71,72,73,74,75,76,77,78,79,80]. Lipid peroxidation activates the nuclear factor kappa-light-chain-enhancer of activated B cells (NF-κB) signaling pathway with the consequent release of different pro-inflammatory cytokines, creating a pro-inflammatory environment that could eventually contribute to AMD progression [81]. Recently, Kim et al. used a murine AMD model (induced by the injection of hydroperoxy-octadecadienoic acid-HpODE, a peroxidized lipid, into the subretinal space) to study the pathologic processes involved in the degeneration of the retina and choroid and demonstrated an early increase in the expression of markers of oxidative stress (and lipid peroxidation in particular as demonstrated by the high levels of 4-HNE and MDA) and inflammation, followed by the infiltration of inflammatory cells into the subretinal space and neural retina and concomitant RPE and photoreceptors damage [82]. The expression of some oxidative stress response genes, such as nicotinamide adenine dinucleotide phosphate (NADPH) oxidases (NOX and DOUX) genes, was upregulated as early as 2 and 5 days after HpODE injection [82].

All these processes trigger the activation of different pathways in many cell types, both of the retina and choroid, involved in chronic inflammation (e.g., through the activation of the NF-κB pathway) [83,84,85,86], impairment of autophagy, activation of the complement system and hypoxia [87,88], with the consequent further release of ROS and the initiation of a vicious cycle and progressive amplification of the pathologic events that result in cell death and AMD progression [83,84,85,86,89]. In ARPE-19 cells, the accumulation of excessive amounts of ROS triggers the activation of the nod-like receptor family pyrin domain containing 3 (NLRP3) inflammasome, an important regulator of the secretion of different pro-inflammatory cytokines, through the increase in lysosomal membrane permeabilization, the activation of the mitogen-activated protein kinase (MAPK) and NF-κB signaling pathways, and probably also local activated microglial cells [90,91,92]. The mechanisms leading to RPE cell death induced by oxidative stress are still poorly understood [93] and, even if apoptosis has been the most studied mechanism of programmed cell death (PCD) in the RPE [94], more recent studies have suggested that other forms of PCD, such as necroptosis (a form of regulated necrosis) and ferroptosis (a form of iron-dependent non-apoptotic programmed necrosis), could be involved in AMD pathogenesis [93,95,96,97,98,99].

### 3.1. The Role of Mitochondria in ROS Production and Oxidative Damage in the Early Phases of AMD

Mitochondria are suggested to play an important role in AMD pathophysiology. High numbers of mitochondria are present in metabolically active cells like RPE cells, while their number decreases with age and disease [100,101]. Decreased mitochondrial number, function and ATP production have been demonstrated in RPE cells isolated from human AMD eyes [102] and previous reports showed mitochondrial depolarization, with reduced energy production and an increase in Cytochrome C release and ROS generation, to precede RPE cell death caused by peroxidation [103,104,105], thus highlighting the existence of a link between mitochondrial impairment, RPE degeneration and an unbalanced cellular redox system. Mitochondrial electron transport chain (ETC) complexes are a major source of ROS as a byproduct of respiration [106,107,108] and, under pathologic conditions involving ETC components, ROS production and leakage into the cytoplasm are significantly increased and not balanced by the local anti-oxidative stress metabolism [109]. Ultrastructural damage, decrease in number and decreased levels of mitochondrial heat shock protein 70 have been observed in mitochondria of eyes with AMD [110,111]. Mitochondrial DNA (mtDNA) is particularly susceptible to oxidative damage due to the absence of protective histones and intrones and to its high transcription rate with a less effective repair system than nuclear DNA [62], and previous studies documented mtDNA early damage in the RPE of patients with AMD compared to age-matched controls [112,113]. mtDNA damage was found in regions encoding for subunits of the ETC (and in particular in genes that code for the complex I-NADH complex, and III-cytochrome complex) and in the D-loop, the site of initiation for mtDNA transcription and replication of a strand [107,113,114,115]. Different hypotheses exist on the possible mechanisms responsible for the progressive accumulation of mtDNA damage in AMD, including decreased activity of antioxidant enzymes and/or mtDNA repair proteins and impaired clearance capacity [116]. Mitochondrial damage leads to the impairment of the process of oxidative phosphorylation (since all genes encoded by mtDNA are involved in oxidative phosphorylation), with a consequent further increase in ROS generation and the initiation of a vicious cycle [117,118]. mtDNA damage was specifically found in RPE cells and not observed in photoreceptors [113], and it was hypothesized that this could be due to the different pathways involved in energy production in the two cell types [116].

### 3.2. Impairment of Autophagy and Oxidative Damage

Autophagy is a lysosome-mediated catabolic process that contributes to cellular homeostasis, degrading and removing organelles such as mitochondria with damaged mtDNA (mitophagy), aggregated proteins, lipid droplets and exogenous pathogens [62,119], and its dysregulation has been demonstrated to increase cellular susceptibility to oxidative stress [120]. Beclin-1 and microtubule-associated protein 1 light chain 3 (LC3) are considered reliable biochemical markers of autophagy activation and are used to evaluate the role of autophagy in AMD [121,122]. The reduced efficiency of autophagy in RPE cells, with the consequent reduced removal of damaged proteins and organelles, has been identified as a crucial event in the initiation of AMD changes in models selectively deficient for autophagy-related genes (such as Atg-5 and 7), with formation and accumulation of drusen-like deposits resembling those seen in dry AMD [120,123,124,125,126]. Lipofuscin is believed to further reduce autophagy efficiency by blocking lysosomal enzymes and increasing lysosomal membrane permeability, with the consequent release of the potentially toxic content of lysosomes (redox-active iron, lytic enzymes) and ROS generation [127]. Further confirmation of the role played by impaired autophagy in AMD came from Golestaneh et al.’s research, in which the authors described an increase in the number of autophagosomes and reduced levels of autophagic flux along with increased production of ROS and impaired mitochondrial activity in cultured hRPE of AMD human donors compared to healthy controls [128]. The autophagy response to oxidative stress dynamically modifies under different conditions; Mitter et al. used ARPE-19 cells exposed to H_2_O_2_ for 3–24 h and 14 days to study the cellular response under both acute and chronic oxidative stress and observed an initial increased autophagic activity followed by a reduction in autophagic flux [120].

## 4. Antioxidant Mechanisms in the Retina

Under physiologic conditions, many enzymatic and non-enzymatic antioxidants act to prevent cellular damage and death induced by ROS accumulation. Under pathologic conditions, the cellular capacity to neutralize ROS decreases due to a loss of efficiency of the antioxidant systems (with reduced production of ROS scavengers and other antioxidants) parallel to an over-production of ROS [112,129]. Brown et al. demonstrated that the lack of the mitochondrial antioxidant enzyme SOD2 in the RPE induced high cellular oxidative stress with consequent RPE and photoreceptor dysfunction [130]. Cellular strategies intended to limit oxidative damage include the restriction of the site and action of oxidant-producing enzymes to regions adjacent to the target, e.g., H_2_O_2_ transport is limited by the presence of aquaporin channels [131]; moreover, ROS have a short half-life and their production is time-limited to the oxidant burst [22]. Moreover, oxidative stress induces the upregulation of specific antioxidant enzymes, such as manganese superoxide dismutase (MnSOD) and catalase as a compensatory response to a highly oxidative environment [132,133]. A number of transcription factors are involved in the regulation of antioxidant systems and, between them, nuclear factor erythroid-2 related factor 2 (Nrf2, also known as NFE2L2) and peroxisome proliferator-activated receptor gamma coactivator-1 (PGC-1) play key roles in the activation of cellular antioxidant defense systems.

### The p62/Keap1/Nrf2 and PGC-1 Pathways

The Nrf2 and PGC-1 pathways are critical pathways in both the activation of autophagy and the protective response to oxidative stress [123]. Nrf2 is a transcription factor that regulates cellular homeostasis, protecting cells from oxidative damage and preventing ROS-induced retinal cell death [134,135,136]. Under physiologic conditions, Kelch-like ECH-associated protein 1 (Keap1) degrades Nrf to prevent its translocation from the cytosol to the nucleus [136]. Under conditions of oxidative stress, Nrf2 translocates into the nucleus and initiates the transcription of antioxidant genes, such as catalase or SOD, and autophagy-related genes [137,138,139,140]. Wang et al. demonstrated that in eyes affected by early AMD, Nrf immunolabeling is decreased in damaged RPE cells and increased in morphologically normal RPE cells, supporting the hypothesis that, under conditions of high oxidative stress, the Nrf pathway is impaired and not sufficient to prevent cellular damage [141]. The development of drusen and RPE changes were demonstrated in knock-out murine models for Nrf, however the impairment of this signaling pathway was not sufficient to induce progression to the advanced stages of disease, such as nAMD [52]. The scaffolding adaptor protein p62/sequestosome 1 (SQSTM1) pathway, specifically involved in the autophagy of protein aggregates and mitophagy and acting as a bridge with the ubiquitin–proteasome system (the other major system of cellular clearance), could activate the Nrf2 pathway by binding to Keap1 and promoting its degradation to block its interaction with Nrf2 [142,143,144], and this further highlights the close interplay between mechanisms involved in autophagy and the response to oxidative stress. PGC-1α, PGC-1β and PGC-1-related coactivators form a defense system against oxidative stress by upregulating the expression of antioxidant genes, such as SOD2 and thioredoxin 1 (TRX1), and the loss of function of this system can contribute to AMD initiation, increasing the formation of ROS by mitochondria and the accumulation of mitochondrial damage [142]. A knock-out murine model for PGC-1α exposed to a high-fat diet developed drusen-like deposits and RPE cell and photoreceptor degeneration, along with evidence of reduced autophagy flux [145], thus implying that the PGC-1 pathway is also involved in autophagy regulation against oxidative damage. Felszeghy et al. used Nrf2/PGC-1α double knock-out mice to study the role of autophagy in the development of dry AMD and observed high levels of oxidative stress markers, such as 4-HNE, damaged mitochondria and large autolysosomes in the RPE cells, along with an increase in p62/SQSTM1, Beclin-1 and LC3B [146]. We already reported evidence on how reduced autophagic activity could lead to cellular damage in AMD in the previous paragraph (see Section 3.2.).

## 5. Oxidative Stress and Late Neovascular AMD

In the previous paragraphs, scientific evidence of the role of oxidative stress as a trigger for the initial stages of AMD was reported and discussed. Even if the exact mechanisms leading to neovascularization in the late stages of AMD are not fully elucidated, it is hypothesized that oxidative stress could be involved even in this case. In the past, it was debated whether oxidative stress could directly promote NV or if it is only involved in drusen formation and progressive RPE, and photoreceptor dysfunction and death, which finally lead to NV [147]. However, recent evidence suggests that the association of oxidative stress, hypoxia and impaired autophagy may be a stimulus for VEGF secretion in RPE cells, thus contributing to induce the development of new vessels, which are the hallmark of nAMD [62,148,149,150]. 

Dong et al. used a murine model deficient in SOD1 to study how a compromised antioxidant defense system could contribute to create a pro-angiogenic environment in the subretinal space, with the consequent development of NV, and they observed that Sod1^−/−^ mice had a significantly lower expression of VEGF and more NV compared to Sod^+/+^ mice [147]. ROS have been shown to induce the expression of VEGF189, a splice variant of VEGF-A, in cultured hRPE cells exposed to H_2_O_2_ [151]. More recently, mitochondrial dysfunction has also been hypothesized to play a role in promoting neovascularization in AMD [152]. Enzymes belonging to the NOX family (NOX1, NOX2 and NOX4 in particular) have been proposed as one connection between ROS generation and VEGF release in the human choroid [153,154]. NOX homologues are major sources of ROS in the vasculature, with the consequent proliferation and migration of endothelial cells, activation of the transcription factor NF-κB and increased expression of VEGF, directly or through hypoxia-inducible factor 1-alpha [154,155,156,157,158]. VEGF in turn can stimulate the generation of ROS through the activation of Rac1, a subunit of NOX, and this interplay creates a vicious cycle that progressively increases the release of both VEGF and ROS leading to endothelial cell proliferation and migration and the formation of new vessels [153,159,160].

Previous reports observed an increased peroxidation activity with disease progression from early stages to nAMD [161]. The activation of 12/15-lipoxygenase, the enzymes catalyzing lipid peroxidation, has been shown to increase ROS generation, the expression of NOX2 and vascular permeability (via activation of the VEGF-R2 signaling pathway) [162]. Recently, Zor et al. measured serum levels of MDA in the samples of 30 patients with nAMD and detected significantly higher values of this biomarker compared to age-matched controls [163], confirming previous studies that observed serum MDA levels 14.9% higher in patients with nAMD compared to controls [80]. Moreover, low-dose MDA treatment for 48 h on ARPE-19 cells was demonstrated to induce VEGF expression and modifications in autophagic activity, whereas the MDA-induced VEGF increase was reduced by 3-methyladenine and ammonium chloride, an autophagy and a lysosomal inhibitor, respectively; therefore, the authors speculated that MDA might act on VEGF secretion through the autophagy–lysosomal pathway [80]. In particular, Bergmann et al. detected a significant increase in the isoforms VEGF 121 and VEGF 165 after the exposure of ARPE-19 cells to MDA or HNE [164]. Ye et al. further investigated the correlation between MDA administration and VEGF secretion in vivo in a murine model, and observed that the injection of MDA in the vitreous cavity of mouse eyes significantly increased VEGF levels in the RPE/choroid and the volume of laser-induced NV compared to controls injected with phosphate-buffered saline [80]. These findings were further confirmed in a following study showing a direct correlation between MDA serum levels and neovascular lesion area in AMD patients [165]. In their murine model to study the effects of oxidative stress (induced by the injection of HpODE, see Section 3) on retinal and choroidal tissues, Kim et al. observed a progressive accumulation of activated microglia and inflammatory cells in the subretinal space from day 5 to day 20, followed, at day 20, by the appearance of new vessels breaking through BrM into the retina, along with a significant increase in VEGF levels in RPE cells and in the choroid [82]. 

Animal models exposed to acute light damage (LD) are also frequently used to study retinal degeneration induced by oxidative stress [166,167], and Tisi et al. recently used this model to investigate the correlation between LD and nAMD in vivo, finding a significant upregulation of VEGF-A and other pro-angiogenic factors (such as basic fibroblast growth factor, bFGF) and subsequent early NV (starting 7 days after light exposure), along with the detection of long-term activated microglia surrounding the new vessels; therefore, they suggested that microglial cells might be implicated in the process of neoangiogenesis in AMD [167]. Other recent studies have postulated that microglia could be a target of oxidative stress, triggering a vicious cycle of inflammation and cellular degeneration [168]; blockade of the microglial adenosine receptor A_2A_ has been associated with a reduction in the levels of ROS and concomitant retinal degeneration, and a dose-dependent increase in microglia activation (along with an increase in pro-inflammatory cytokines, inducible nitric oxide synthase and HSP70) was observed after injection of H_2_O_2_ in an in vitro retinal model [169,170].

## 6. Nitric Oxide and Nitrosative Stress Role in AMD

Nitric oxide (NO) is a gas-signaling molecule synthesized as a byproduct of the reaction of the conversion of L-arginine to L-citrulline catalyzed by three different isoforms of NO synthase (NOS) [171]: the constitutive and calcium-dependent neuronal NOS (nNOS), the inducible and calcium-independent NOS (iNOS) and the endothelial NOS (eNOS) isoforms [172]. While the nNOS and eNOS isoforms are constitutively expressed and play a role as regulators of physiological phenomena, the inducible isoform iNOS is thought to be involved in cytotoxic and inflammatory functions [173,174,175]. Therefore, NO could exert cellular protective or harmful effects depending on its concentration and on the balance between the activity of constitutive/inducible isoforms of NOS [174].

In the retina, NOS isoforms can be found in retinal neurons, RPE, amacrine and ganglion cells, nerve fibers and photoreceptors and evidence has emerged about the role of NO as a mediator of physiological, and possibly pathological, processes in all the above-mentioned retinal structures [174,176]. In this context, small amounts of eNOS-derived NO could act as a potent vasodilator and play a key role in the physiological regulation of ocular blood flow; furthermore, in the RPE, NO contributes to the function of the phagocytosis of rod outer segments and to the regulation of VEGF gene expression [177,178,179]. However, under conditions of oxidative stress, reactive nitrogen species (RNS), such as peroxynitrite, nitrogen dioxide, dinitrogen trioxide and peroxynitrous acid, produced by high levels of NO, can induce cellular damage as a result of reactions with proteins and DNA [180,181]. In particular, NO in high concentrations reacts with superoxide anions (O_2_^•−^) to produce peroxynitrite (ONOO^−^), a potentially cytotoxic tyrosine-nitrating molecule, which could induce the accumulation of protein aggregates between photoreceptors and RPE cells, with final photoreceptor degeneration [177,178,179]. Even if the exact role of NO in AMD pathogenesis is still not clear, recent studies have hypothesized that changes in NO levels and NOS activation/expression in AMD patients could be involved in the modulation of choroidal perfusion, which could contribute to the development of AMD [177].

### Nitric Oxide and Nitrosative Stress in Late Neovascular AMD

**Figure 3:**
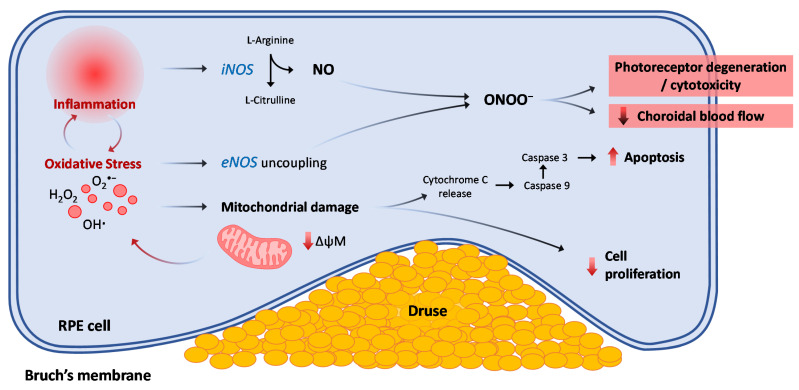
Schematic representation of the intracellular mechanisms leading to progressive changes observed in AMD as a consequence of oxidative and nitrosative stress. iNOS: inducible nitric oxide synthase; eNOS: endothelial nitric oxide synthase isoform; ONOO^−^: peroxinitrites; ROS: reactive oxygen species; ΔψM: mitochondrial membrane potential; AMD: age-related macular degeneration.

An important role of NO in the late stages of AMD, in particular in nAMD, has been documented, as nitrosative stress is thought to play a role in the microcirculatory changes observed in the choroid of patients with nAMD [175,182]. The large amount of iNOS-derived NO could be, at least in part, responsible for the vascular damage in the retina observed in AMD, as a consequence of increased peroxinitrite generation [174]. The imbalance between NO and peroxinitrites could affect choroidal perfusion by both reducing the availability of NO and increasing RNS production, with consequent vasoconstriction and cellular damage. Moreover, it has been demonstrated that, under pathologic conditions, oxidative stress is able to convert eNOS from a NO-producing enzyme to a superoxide-producing enzyme in a reaction that causes the reduction in glutathione levels (a process known as NOS uncoupling), thus turning from being a “beneficial” NOS isoform to a detrimental one, and further contributing to increase cellular oxidative damage (Figure 3) [183,184]. In the retina, eNOS is constitutively expressed in the endothelial cells and smooth muscle cells of retinal arteries and capillaries, but its presence has also been detected in photoreceptors, horizontal cells, bipolar and ganglion cells, amacrine and Müller cells [177,185,186], nNOS in the outer and inner plexiform layers and in bipolar, amacrine and ganglion cells [177,187,188,189,190]. Both eNOS and nNOS isoforms have been shown to play an important role in physiologic ocular functions (such as phototransduction and the regulation of retinal blood circulation) and in the onset of retinal diseases [174,191,192,193]. iNOS expression is induced in the inner retina and outer segments of photoreceptors by inflammatory conditions [194,195,196]. In the choroid of aged healthy subjects, eNOS was found to be prevalently expressed in the CC, while nNOS was expressed in the nuclei of RPE cells and in perivascular nerve fibers surrounding arteries and arterioles [177]. Bhutto et al. studied the localization of NOS isoforms in 22 human donor eyes with AMD (both non-neovascular and nAMD) by immunohistochemistry and compared their distribution to that observed in age-matched controls; they detected a significant decrease in nNOS and eNOS in the retina and choroid of AMD patients and postulated that reduced concentrations of NO may be associated with vasoconstriction and blood flow decrease (secondary to the loss of CC and perivascular nerve fibers) in the submacular choroid in AMD [177]. In the same study, the authors performed a sub-analysis on eyes with nAMD, investigating the differences in NOS isoform expression in the area occupied by new vessels and in the underlying and adjacent choroid. They observed high levels of eNOS and iNOS in correspondence with new vessels and a low expression in the underlying and adjacent choroid, thus confirming the involvement of NO in nAMD [177]. Previous studies demonstrated that NO is a mediator of angiogenesis through the regulation of different proangiogenic factors, including VEGF [197], and the VEGF proangiogenic effect requires the activation of eNOS with the consequent release of NO [198,199,200]. It has also been shown that iNOS could be a promoter of neovascularization and, based on the observations on a murine model knock-out of the NOS gene and on cell cultures with a down-regulated iNOS/NO/VEGF signaling pathway, researchers suggested that inhibition of nNOS and iNOS expression may reduce the rate of formation of MNV [201,202,203]. Therefore, it was hypothesized that NOS activity may induce NV through increased VEGF expression under hypoxic conditions [177,193].

## 7. The Role of Anti-VEGF Agents against Oxidative/Nitrosative Stress

**Figure 4:**
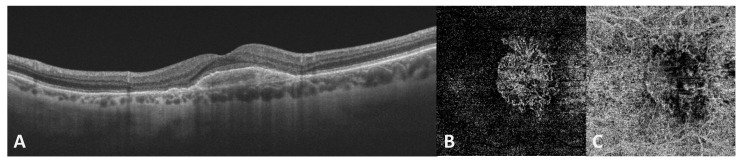
Left eye of a 72-year-old female patient with neovascular AMD previously treated with 10 intravitreal injections of anti-VEGF. (**A**) Optical coherence tomography (OCT) B-scan centered on the fovea showing a subfoveal fibrovascular PED with no signs of active exudation. (**B**,**C**) OCT-angiography (OCT-A) 4.5 × 4.5 mm macular slab segmented at the outer retina (**B**) and choriocapillaris (**C**) level showing the presence of MNV. AMD: age-related macular degeneration; PED: pigment epithelium detachment; MNV: macular neovascularization.

Anti-VEGF agents are currently the standard of care for exudative nAMD (Figure 4) [204]; however, their role against oxidative damage is still poorly investigated. 

In a recent study, our group examined the effects of Aflibercept and Ranibizumab on oxidative stress in vitro on cultured ARPE-19 cells and showed that both agents (used at the same concentrations obtained in the human eye after intravitreal injection) were positively involved in the modulation of cell viability and mitochondrial function in RPE cells, acting on mechanisms mediated by NO release and the regulation of autophagy both under physiologic conditions and after exposure to H_2_O_2_ [205]. In particular, under conditions of oxidative stress, Aflibercept and Ranibizumab were able to reduce NO release induced by H_2_O_2_, by modulating the activity or the expression of the eNOS and iNOS isoforms [205]. For the reasons explained in the previous paragraph (see Section 6), balancing NO release could be helpful to preserve choroidal blood flow, regulate the process of the phagocytosis of photoreceptor outer segments and reduce peroxinitrite generation and release.

**Figure 5:**
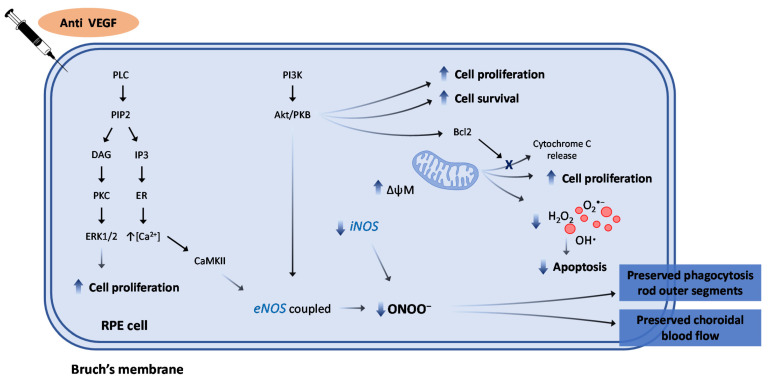
Schematic representation of the intracellular pathways activated by anti-VEGF agents in AMD. PLC: phospholipase C; PIP2: phosphatidylinositol 4,5-bisphosphate; DAG: diacyl glycerol; IP3: inositol triphosphate; PKC: protein kinase C; ER: endoplasmic reticulum; PI3K: phosphoinositide 3-kinase; PKB: protein kinase B; ERK1/2: extracellular signal-regulated kinase 1/2; CaMKII: calcium/calmodulin-dependent protein kinase II; eNOS: endothelial nitric oxide synthase isoform; iNOS: inducible nitric oxide synthase; ONOO^−^: peroxinitrites; ΔψM: mitochondrial membrane potential.

Contradictory evidence exists in the literature on the effects of anti-VEGFs on mitochondrial function; in fact, while Malik et al. did not find any beneficial effect on mitochondria at clinical doses (and detrimental effects at higher doses) [206], Sheu et al. proposed that anti-VEGF agents could be protective in mitochondria, preserving their energetic metabolism, and might consequently reduce cellular damage caused by oxidative stress [207]. The findings by our group confirmed this second hypothesis, demonstrating a positive effect of both Aflibercept and Ranibizumab on mitochondrial membrane potential, accompanied by the inhibition of apoptosis as shown by the reduction in apoptotic markers, such as Cytochrome C and Caspase 9 [205]. As previous data have shown a clear association between RPE dysfunction and compromised mitochondrial function (see Section 3.1.), the positive effect exerted by anti-VEGFs on mitochondrial membrane potential could be of significant clinical relevance. In the same study, the presence of 3 methyladenine, an inhibitor of autophagy, reduced the positive effect of anti-VEGFs on mitochondrial membrane potential, whereas rapamycin, an activator of autophagy, potentiated this effect, thus suggesting that the beneficial action of Aflibercept and Ranibizumab on mitochondria and cellular survival may be mediated, at least to some extent, by the autophagy pathway [205]. It is also noteworthy that both anti-VEGF agents, in addition to preventing the fall of cell viability and preserving mitochondrial membrane potential, were also able to increase cell proliferation and migration [205] (Figure 5), in contrast with some in vivo studies reporting evidence that anti-VEGF treatment can potentially increase the rate of development of macular atrophy in AMD, though data in the literature on this topic are controversial and not conclusive [208,209,210,211,212,213]. Changes in the ocular microenvironment or effects of anti-VEGF agents on retinal cells other than RPE, as well as in their cross-talk in vivo, could be at the basis of these discrepancies. Therefore, the effects of anti-VEGFs on co-cultures of ARPE-19 cells and human umbilical vascular endothelial cells (HUVEC) were investigated in a following study; this model was created in order to examine the cross-talk between RPE and vascular endothelial cells in the maintenance of the external blood retinal barrier, under conditions of oxidative stress induced by treatment with H_2_O_2_ [214]. The results obtained showed that both Aflibercept and Ranibizumab inhibited the release of NO and ROS induced by H_2_O_2_ and modulated the activation and expression of eNOS and iNOS isoforms in RPE cells; moreover, the presence of a NOS inhibitor decreased the protective effects elicited by anti-VEGFs on RPE cells. Those findings further supported the hypothesis that NO might play an important role as a mediator of the protective effect of the anti-VEGFs on cellular survival [214].

## 8. Conclusions

In the present review, we have highlighted the different pathways involved in the initiation of AMD and in the progression to the late stages of disease in the context of oxidative stress. A growing body of scientific evidence supports the hypothesis that oxidative damage plays a key role in the initiation of AMD pathologic processes, such as drusen formation and RPE changes, with secondary activation of the inflammatory cascade and of mechanisms of cell death. Moreover, the idea that oxidative stress is also directly involved in the process of neovascularization in the late stages of the disease is increasingly gaining ground between researchers. NO contributes to the regulation of choroidal perfusion and the imbalance between NO and RNS observed under pathologic conditions is thought to induce microcirculatory changes in the choroid, both in non-neovascular and nAMD. Anti-VEGF agents have been shown to act against oxidative damage and to have a protective effect on cellular survival by preserving mitochondrial function, the activation of the autophagy defense system and mechanisms mediated by the release of NO. Further research is needed to study new treatments or implement current treatments, with the purpose of better counteracting the damaging effect of oxidative and nitrosative stress in AMD.
